# Effect of Hypoxia on the Differentiation and the Self-Renewal of Metanephrogenic Mesenchymal Stem Cells

**DOI:** 10.1155/2017/7168687

**Published:** 2017-01-17

**Authors:** Shaopeng Liu, Nana Song, Jianqiang He, Xiaofang Yu, Jia Guo, Xiaoyan Jiao, Xiaoqiang Ding, Jie Teng

**Affiliations:** ^1^Division of Nephrology, Zhongshan Hospital, Fudan University, Shanghai 200032, China; ^2^Shanghai Key Laboratory of Kidney and Blood Purification, Shanghai 200032, China; ^3^Shanghai Institute of Kidney and Dialysis, Shanghai 200032, China; ^4^Department of Nephrology, The Affiliated Hospital of Jiangsu University, Zhenjiang, Jiangsu 212001, China

## Abstract

Hypoxia is an important and influential factor in development. The embryonic kidney is exposed to a hypoxic environment throughout its development. The Wnt/*β*-catenin pathway plays vital roles in the differentiation and self-renewal of metanephrogenic mesenchymal stem cells (MMSCs) from which the kidney is derived. Thus, we hypothesized that hypoxia can regulate the differentiation and pluripotency of MMSCs through the Wnt/*β*-catenin pathway. To test this hypothesis, MMSCs from rats at embryonic day 18.5 were cultured in normoxic (21% O_2_) and hypoxic (1% O_2_) conditions. The effects of hypoxia on differentiation, stemness, proliferation, and apoptosis of cultured MMSCs and on the activity of the Wnt/*β*-catenin pathway were tested. Our results revealed that the hypoxic condition increased the number of epithelial cells (E-cadherin^+^ or CK18^+^) as well the expression of markers of renal tubule epithelia cells (CDH6, Aqp1, and OPN), decreased the number and proliferation of stem cells (SIX-2^+^ or CITED1^+^), and induced apoptosis. Additionally, hypoxia reduced the expression of Wnt4 as well as its downstream molecules *β*-catenin, LEF-1, and Axin2. Activation of the Wnt/*β*-catenin pathway by LiCl or BIO modified the effects of hypoxia on the differentiation and self-renewal of MMSCs. Thus, we concluded that hypoxia induces the differentiation and inhibits the self-renewal of MMSCs by inhibiting the Wnt/*β*-catenin pathway. The observations further our understanding of the effects of hypoxia on kidney.

## 1. Introduction

Prenatal hypoxia is a common complication during pregnancy, which leads to negative foetal outcomes such as birth defects [1]. It has been reported that foetal hypoxia decreases nephron numbers and kidney weight [2]. However, it has been shown that hypoxia stimulates ureteric bud branching in vitro [3]. In this regard, an appropriately low O_2_ tension is necessary for embryonic development. Because the effect of hypoxia on development of the kidney is controversial, understanding the kidney's responses to oxygen deprivation is critical.

Coordinated interactions between the embryo and its environment are critical for development. Early-stage mammalian embryos develop in hypoxic environments and live at low oxygen concentrations (ranging from 2% to 9%) throughout foetal development [4, 5]. A hypoxic environment represents the physiological growing conditions of embryonic stem cells. Many studies have indicated that hypoxia influences development of the embryo by regulating the differentiation and self-renewal (including the maintenance of stemness and proliferation) of stem cells. In some organs, such as the lung, nervous system and heart, hypoxia induces the differentiation of stem cells into mature cells [6–8]. For other cells such as cochlear spiral ganglion stem cells, human mesenchymal stem cells, and adipose-derived mesenchymal stem cells, hypoxia helps to maintain stemness to preserve the stem cell pool [9–11]. The kidney can exist at 1% O_2_ or even lower oxygen tension, owing to atypical blood vessel networks [12]. Oxygen tension may have a profound influence on the development of the kidney.

The development of the mammalian kidney is guided by reciprocal inductive interactions between the ureteric bud (UB, giving rise to the collecting duct system) and the metanephric mesenchyme (giving rise to all other renal epithelial cells) [13]. In rats, the metanephric kidney develops at embryonic day 12, and this is followed by the ingrowth of the UB into the metanephric blastema, inducing the metanephric mesenchymal stem cells (MMSCs) at the bud tips to condense around the UB tips. Subsequently, the condensed cells undergo a mesenchymal-epithelial transition (MET), thus forming epithelial vesicles, and this is followed by sequential differentiation into functional nephrons [14, 15]. The balance between the differentiation and self-renewal of MMSCs is essential for development of the kidney. However, the effects of hypoxia on the differentiation and self-renewal of MMSCs have been unclear.

Among many other signalling pathways, the Wnt/*β*-catenin pathway is considered to play a critical role in development of the kidney. Upregulation of Wnt4, which mediates epithelialization of the metanephric mesenchyme, has been detected after exposure of mesenchymal stem cells to hypoxia [16]. Disruption of Wnt4 impairs MET in the embryonic kidney; conversely, coculture of the mesenchyme with cells expressing Wnt4 induces tubulogenesis in organ culture [17, 18]. In contrast, inhibition of *β*-catenin (downstream of Wnt pathway) induces tubulogenesis [19] and constitutive expression of stabilized *β*-catenin blocks MET in cultured MMSCs [20, 21]. Thus, we hypothesized that hypoxia may regulate the differentiation and self-renewal of MMSCs by a Wnt4-dependent pathway. To test this hypothesis, we used hypoxic culture of isolated MMSCs to address the effects of hypoxia on development of the kidney. In embryonic kidney, the transcription factor sine oculis homeobox homolog 2 (SIX-2) and aspartic acid rich C terminal domain 1 (CITED1) that are essential for self-renewal are considered as markers of MMSCs [14, 22]. E-cadherin and cytokeratin 18 (CK18) are markers of mature epithelial cells; K- cadherin (CDH6) is an early marker of epithelial commitment of nephron progenitors; aquaporin 1 (Aqp1) and osteopontin (OPN) are a marker of renal tubule epithelia cells [23–26]. Our results demonstrated that the number of E-cadherin- or CK18-positive cells was increased, the expression of CDH6, Aqp1, and OPN was elevated, and the number of SIX-2- or CITED1-positive cells was decreased in hypoxia-exposed MMSCs. Additionally, proliferation of MMSCs was inhibited, and apoptosis was promoted by hypoxia. This means that hypoxia promoted the differentiation and inhibited the self-renewal of MMSCs. Furthermore, we found that hypoxia inhibited the Wnt4/*β*-catenin pathway. Activation of Wnt/*β*-catenin with lithium chloride (LiCl) or 6-bromoindirubin-3-oxime (BIO) attenuated the effect of hypoxia. Thus, we conclude that hypoxia promotes the differentiation and depresses the self-renewal of MMSCs by inhibiting the Wnt4/*β*-catenin pathway.

## 2. Materials and Methods

### 2.1. Animals

Twenty timed-pregnant female Sprague-Dawley rats were provided by the Animal Center of Shanghai Medical College, Fudan University (Shanghai, China). At embryonic days 18-19, the age of the embryos was counted from the day of the vaginal plug. This study was performed in strict accordance with the Guidelines on the Care and Use of Laboratory Animals issued by the Chinese Council on Animal Research and the Guidelines of Animal Care. The protocol was approved by the Committee on the Ethics of Animal Experiments of Fudan University.

### 2.2. Metanephric Mesenchymal Stem Cell (MMSC) Culture

The primary MMSC cultures were prepared as previously described [27]. Briefly, rat embryonic kidney rudiments were microdissected at embryonic days 18-19 (E18-E19). The ureteric bud was removed from isolated rudiments, and the remaining mesenchyme was incubated in 0.05% trypsin/0.5 mM EDTA for 5 minutes on ice. Foetal calf serum was added at a concentration of 50% to inactivate trypsin. The suspension was mechanically dissociated by gentle aspiration with a Pasteur pipette. The single-cell suspension was centrifuged at low speed (1500 rpm) for 5 minutes and plated at densities of 2 × 10^5^ cells/ml on 30 mm culture dishes or 96-well plates or 3-well chamber slide system in Dulbecco's modified Eagle's medium (DMEM)/F12 (1 : 1) including 10% foetal calf serum and grown at 37°C, 5% CO_2_, and 95% air (approximately 21% O_2_). After attachment, cells were treated with 1% O_2_ in a variable oxygen control incubators (Thermo Scientific) which could generate hypoxic conditions and monitor real-time O_2_ sensitively or maintained at 21% O_2_ for 3 days. To activate the Wnt/*β*-catenin pathway, LiCl (20 mM) or BIO (5 nM) was added to the culture medium.

### 2.3. Western Blotting

Western blotting was performed to examine CITED1, SIX-2, E-cadherin, and the related proteins in the Wnt/*β*-Catenin signalling pathway (Wnt4, *β*-catenin, and LEF1). Briefly, after culture under hypoxic or normoxic conditions for 3 days, MMSCs were collected, and protein was extracted. The proteins were separated by 12.5% SDS–PAGE, electrotransferred to a PVDF membrane (Millipore, USA), and subsequently blocked with 5% (w/v) fat-free milk in TBST for 1.5 h at room temperature. The membranes were blotted sequentially with primary antibodies, including anti-GAPDH antibody (1 : 5000, Sigma-Aldrich, Catalog number SAB2701825), anti-SIX-2 antibody (1 : 1000; Proteintech, Catalog number 11562-1-AP), anti-CITED1 antibody (1 : 1000; Abcam, Catalog number ab213429), anti-E-cadherin antibody (1 : 5000; BD, Catalog number 610181), anti-Wnt4 antibody (1 : 600; Abcam, Catalog number ab91226), anti-*β*-catenin antibody (1 : 1000; Proteintech, Catalog number 51067-2-AP), anti-LEF1 antibody (1 : 1000; Cell Signalling Technology, Catalog number 2230), and anti-HIF1*α* antibody (1 : 1000; Cell Signalling Technology, Catalog number 14179), and then with secondary antibodies (1 : 5000; Sigma-Aldrich, Catalog numbers RABHRP1 and RABHRP2) conjugated with horseradish peroxidase. The last step of western blotting antibody detection was performed with western blot chemiluminescent HRP substrate reagent (Thermo Fisher). The expression levels of different proteins were normalized to those of the internal control (GAPDH) [28].

### 2.4. Real-Time PCR

The total RNA was isolated from MMSCs using TRIzol (Life Technologies, USA). The RNA levels of SIX-2, Wnt4, LEF1, Axin2, PKG1, Glut1, CDH6, Aqp1, and OPN were detected with a SYBR-Green RT-qPCR kit (Takara, Japan) according to the manufacturer's instructions (Sangon Biotech, China). The levels of mRNA expression were normalized to those of the housekeeping gene (GAPDH). Simple relative quantification of target gene expression normalized to GAPDH was performed using the 2^−ΔΔCt^ method [29]. The primer sequences are shown in Table 1.

### 2.5. Flow Cytometry

Cells were harvested from the culture dishes using trypsin EDTA and fixed with 4% paraformaldehyde, pH 7.4, dissolved in PBS. Single cells for flow cytometry (FCM) were incubated for 30 min with their respective primary antibodies (1 *μ*l for 10^6^ cells, CK18: Abcam, Catalog number ab82254) and subsequently washed with PBS supplemented with 1% FBS. After removing the primary antibody by washing twice, samples were reincubated with either a FITC-, Cy3, or Alexa flour 594-conjugated secondary antibody at a dilution of 1 : 500 using an exposure time of 20 min. Apoptosis was detected using Annexin V-FITC/PI staining kit (Beyotime Institute of Biotechnology, China) according to the manufacturer's instructions. Before starting FCM, cells were again rinsed twice with wash buffer. FCM was carried out using a FACSCalibur (Becton Dickinson, Heidelberg, Germany).

### 2.6. Immunofluorescence

The MMSCs (2 × 10^5^ cells/ml in 500 *μ*l complete medium) were seeded into each well of chamber slide system. After 24 hours of normoxic incubation, the chamber was removed, and cells were fixed with methanol for 10 minutes. The cells were preincubated with 5% normal goat serum to block nonspecific binding and then incubated with primary antibodies (1 : 100 rabbit anti-SIX-2 or CITED1 and 1 : 100 mouse anti-*α*-SMA, Sigma-Aldrich, Catalog number A5228) at 4°C overnight. Extensive washing was followed by incubation with FITC- or Cy3-conjugated secondary antibody (1 : 200) for 1 h at room temperature. Slides were mounted with antifade mounting medium (Beyotime Institute of Biotechnology, China) with DAPI (4′,6-diamidino-2-phenylindole). Images were acquired with a confocal laser scanning microscope (Zeiss 510).

### 2.7. TUNEL and EdU Staining

The MMSCs (2 × 10^5^ cells/ml in 500 *μ*l complete medium) were seeded into each well of chamber slide system and incubated under hypoxic or normoxic condition for 3 days. Apoptosis was detected using a TUNEL apoptosis assay kit (KeyGen Biotech, China). Cells growing on slides were fixed in 1% formaldehyde for 30 min, permeabilized (using 1% Triton-X100 in PBS) for 10 min, and rewashed before application of TUNEL reagents diluted to 50% with TUNEL dilution buffer. After washing, positive staining was detected with label solution. Proliferation of cells was detected using keyFluor488 Click-iT EdU kit (Beyotime Institute of Biotechnology, China). Cells were incubated with 10 *μ*M EdU for 1 hour prior to being harvested and then fixed and permeabilized as described above. For EdU staining, the slides were incubated with Click-iT reaction cocktail containing Alexa Fluor 488 for 30 min at room temperature according to the manufacturer's instructions.

### 2.8. Cell Viability Assay

The effect of hypoxia on the viability of MMSCs was determined using AlamarBlue cell viability assay kit (Beyotime Institute of Biotechnology, China) as previously described. Briefly, the MMSCs (1 × 10^4^ cells/well in 100 *μ*l complete medium) were seeded into 96-well cell plates. After 72 hours of hypoxic or normoxic incubation, 10 *μ*l AlamarBlue ready-to-use solution was added to each well and the plates were incubated at 37°C for a further 2 hours. The absorbencies with wavelengths set at 570/600 nm were measured by SpectraMax fluorescence multiwell plate reader. Breeding ratio (%) = (117216 ×* A*570 sample − 80586 ×* A*600 sample)/(117216 ×* A*570 control − 80586 ×* A*600 control) × 100%.

### 2.9. Statistical Analysis

Data are presented as means ± SEM and were analysed using SPSS software. Statistical analyses including the independent *t*-test (comparison of two groups) and one-way ANOVA followed by Tukey's post hoc test (comparison of more than two groups) were regarded as significant when *p* < 0.05.

## 3. Results

### 3.1. Characterization of the Isolated MMSCs

**Figure 1:**
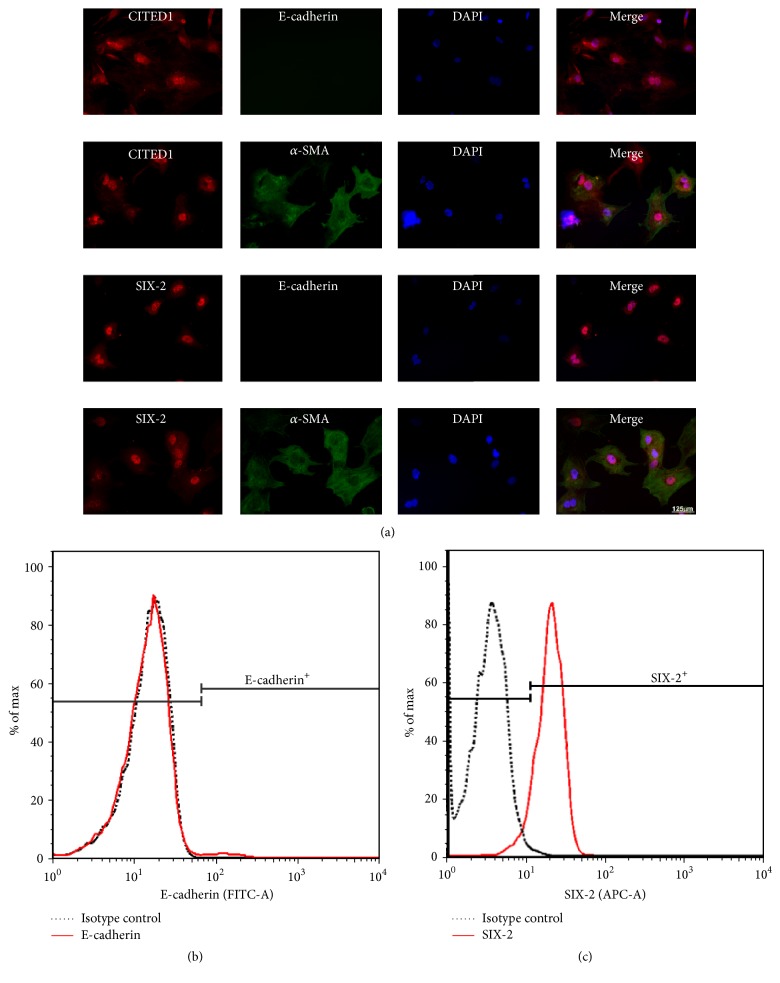
The isolated cells were identified as MMSCs by immunofluorescence double staining and flow cytometry. (a) The isolated cells were plated on chamber slide system and cultures in 21% O_2_ for 24 h, and then the cells were stained by immunofluorescence. The isolated cells coexpressed SIX-2 or CITED1 (markers of metanephric stem cell) and *α*-SMA (marker of mesenchymal cells). Both SIX-2 and CITED1 were located in the nucleus. *α*-SMA was expressed in cytoplasm. No E-cadherin (marker of epithelium) positive cells were observed. Bar is equal to 125 *μ*m. (b)-(c) As the flow cytometry results shown, the percentage of E-cadherin cells is less than 1% and that of SIX-2 positive cells is over 90%. These results confirmed that the isolated cells were MMSCs.

To verify that the cells isolated using our methods were MMSCs, immunofluorescence double staining was applied to test coexpression of stem cell and mesenchymal cell markers in these cells. Coexpression of stem cell markers (CITED1 and SIX-2) with a mesenchymal cell marker (*α*-SMA) was detected in most of isolated cells. In contrast, no significant expression of the epithelial cell marker (E-cadherin) was observed (Figure 1(a)). This result was confirmed by flow cytometry: the percentage of E-cadherin positive cells among the fresh isolated MMSCs was less than 1%, while percentage of SIX-2 positive cells was more than 90% (Figures 1(b) and 1(c)). Therefore, our results underline that the cells isolated from rat embryo kidney tissue represented specific metanephric mesenchymal stem cells.

### 3.2. Effect of Different O_2_ Tension on the Differentiation and Stemness of the MMSCs

**Figure 2:**
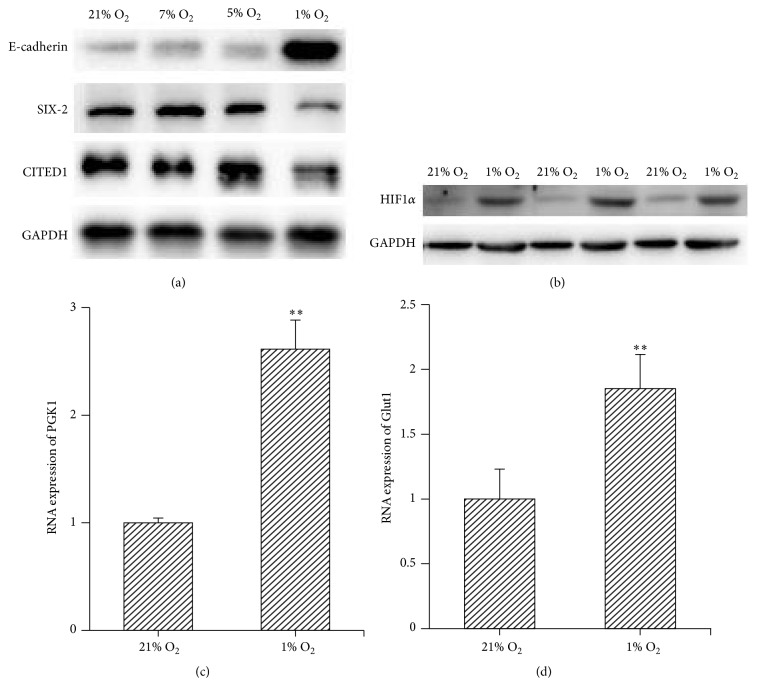
Effect of different O_2_ tensions on the differentiation and stemness of the MMSCs. (a) The MMSCs were cultured under 1%, 5%, 7%, and 21% O_2_ conditions for 3 days, respectively. The expression of E-cadherin, SIX-2, and CITED1 was measured by western blotting. The results show that the expression of E-cadherin, SIX-2, and CITED1 was not altered by 5% or 7% O_2_, compared with 21% O_2_. However, the expression of E-cadherin was increased and the levels of SIX-2 and CITED1 were decreased by 1% O_2_ culture. (b)–(d) The hypoxia environment under 1% O_2_ culture was confirmed by the elevated expression of HIF1*α*, PGK1, and Glut1; ^*∗∗*^*p* < 0.01; *n* = 3.

To select a proper O_2_ tension to investigate the effect of hypoxia on the MMSCs and its underline mechanism, we cultured the fresh isolated MMACs under 1%, 5%, 7%, and 21% O_2_ conditions for 3 days, respectively. The expression of E-cadherin, SIX-2, and CITED1 was measured by western blotting. The results show that the expression of E-cadherin, SIX-2, and CITED1 was not altered by 5% or 7% O_2_, compared with 21% O_2_. However, the expression of E-cadherin was increased and the levels of SIX-2 and CITED1 were decreased by 1% O_2_ culture (Figure 2(a)). Additionally, the hypoxia condition was confirmed by the elevated expression of hypoxia-inducible factors 1*α* (HIF1*α*) and its targets genes PKG1 and Glut1 under 1% O_2_ condition (Figures 2(b)–2(d)). Thus, we considered 1% O_2_ is optimal to research the effect of hypoxic condition on the MMSCs.

### 3.3. Hypoxia Inhibited the Wnt4/*β*-Catenin Pathway

**Figure 3:**
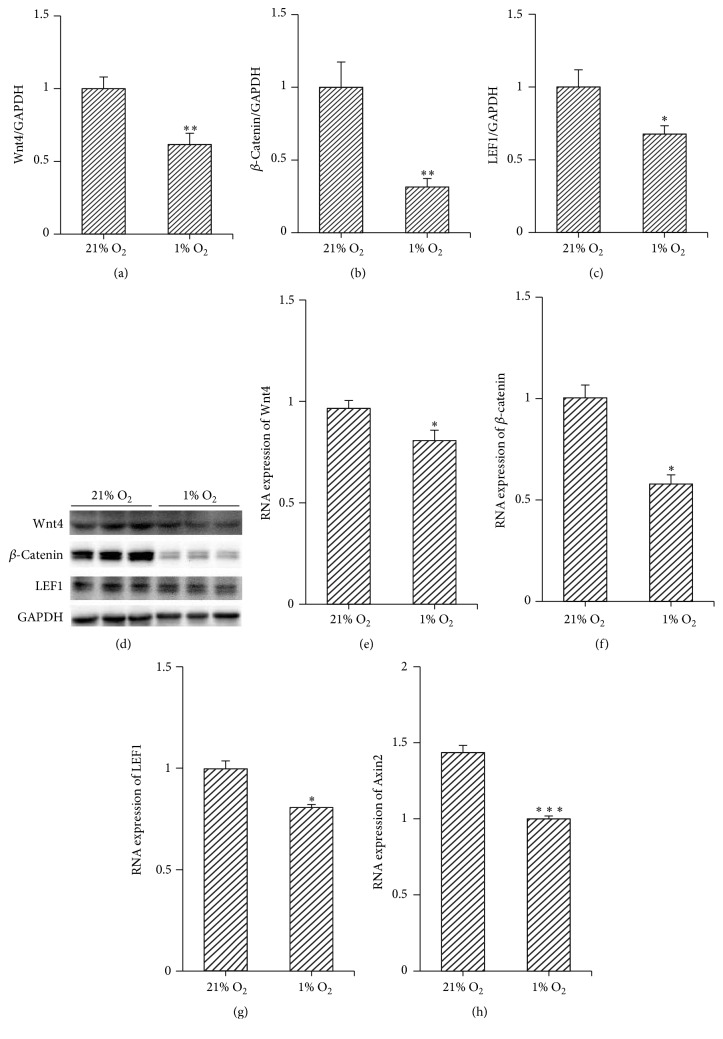
Hypoxia inhibited Wnt/*β*-catenin pathway. Expressions of Wnt4 and downstream molecules in the Wnt pathway, *β*-catenin, LEF1, and Axin2, were measured by western blotting and/or RT-qPCR, 3 days after hypoxic or normoxic culture. Hypoxia decreased the expression of these molecules. (a)–(d) Protein levels of Wnt4, *β*-catenin, and LEF1 were decreased by hypoxic culture; ^*∗*^*p* < 0.05; ^*∗∗*^*p* < 0.01; *n* = 6. (e)–(h) RNA levels of Wnt4, *β*-catenin, LEF1, and Axin2 were also reduced by hypoxic culture; ^*∗*^*p* < 0.05; ^*∗∗∗*^*p* < 0.001; *n* = 3. Data are represented as mean ± SD.

The Wnt signalling pathway is an essential intercellular pathway controlling differentiation. To identify the effect of hypoxia on the Wnt signalling pathway, the expression of Wnt4 mRNA as well as of *β*-catenin, transcription factor LEF1, and target gene Axin2 downstream of the Wnt signalling pathway was analysed by western blotting and/or real-time quantitative PCR. The results showed that both protein and mRNA levels of Wnt4, *β*-catenin, and LEF1 were decreased in 3 days after hypoxia culture (Figures 3(a)–3(g)). Additionally, RNA levels of Axin2 were also attenuated by 3 days of hypoxic culture (Figure 3(h)). These data revealed that hypoxia inhibited the Wnt4/*β*-catenin pathway.

### 3.4. Treatment with Either LiCl or BIO Activated the Wnt/*β*-Catenin Pathway

**Figure 4:**
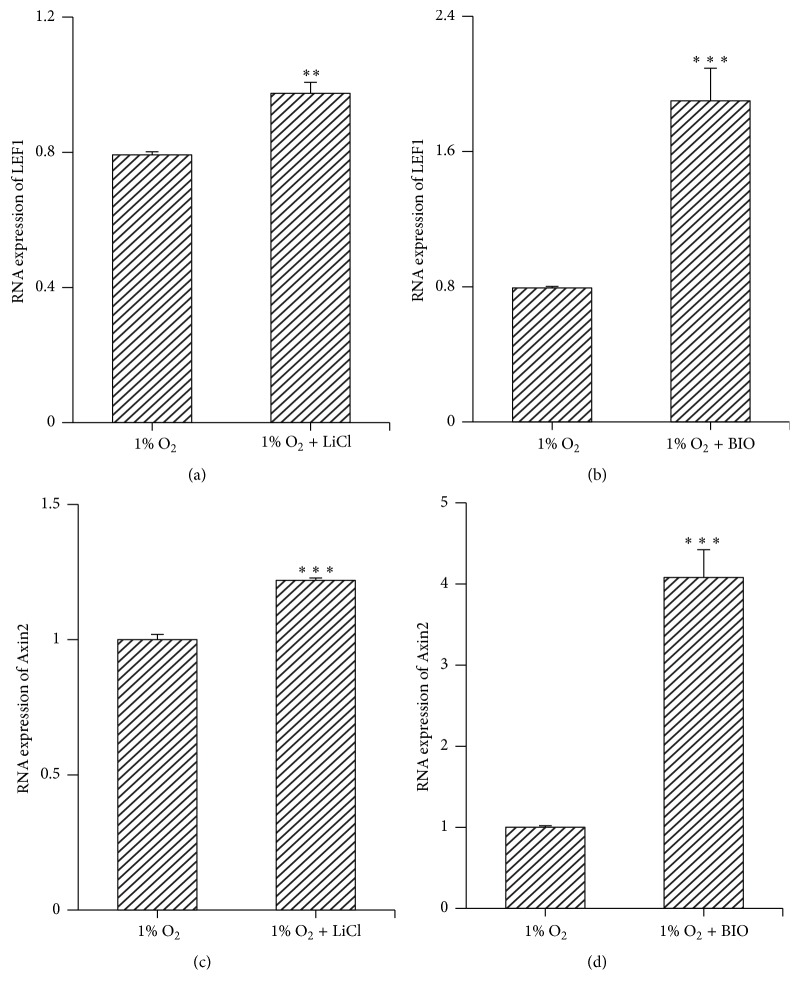
The Wnt/*β*-catenin pathway was stimulated by either treatment of LiCl or BIO. Cells were cultured in complete medium with LiCl (20 mM) or BIO (5 nM) and exposed to hypoxic conditions for 3 days. The expression of LEF-1 and Axin2 was measured by RT-qPCR. (a)–(d) Either LiCl or BIO increased the RNA levels of LEF-1 and Axin2, which suggested stimulation of the Wnt/*β*-catenin pathway. ^*∗∗*^*p* < 0.01; ^*∗∗∗*^*p* < 0.001; *n* = 3. Data are represented as mean ± SD.

To evaluate the role of the Wnt/*β*-catenin pathway in mediating the effects of hypoxia, MMSCs were treated with Wnt pathway activators LiCl or BIO and subjected to hypoxia. After treatment with either LiCl (20 mM) or BIO (5 nM), the observed upregulation of LEF1 and Axin2 suggested activation of the Wnt signalling pathway (Figure 4).

### 3.5. Hypoxia Stimulated the Differentiation of MMSCs by Inhibiting the Wnt/*β*-Catenin Pathway

#### 3.5.1. Hypoxia Stimulated the Differentiation of MMSCs

**Figure 5:**
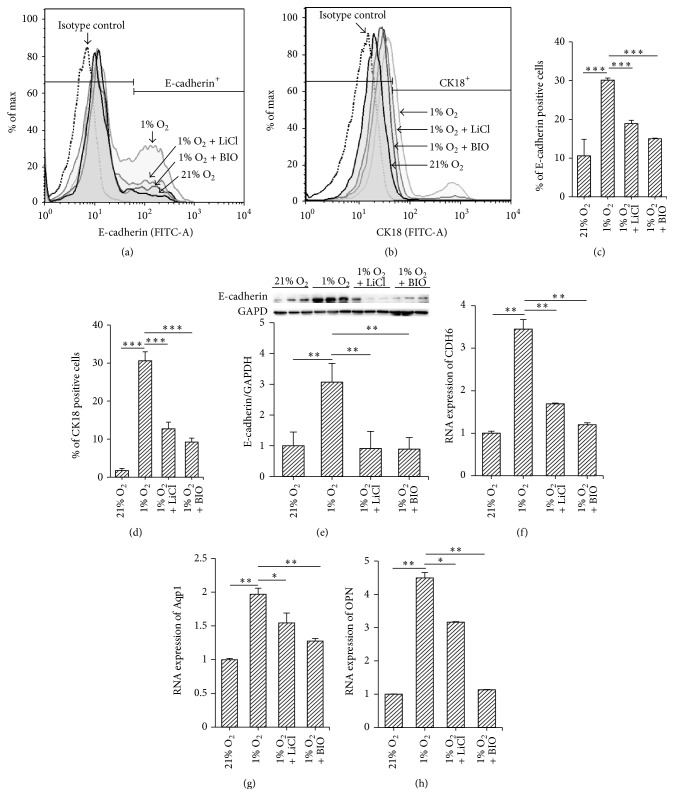
Stimulation of the Wnt/*β*-catenin pathway inhibited the differentiation of hypoxia-conditioned MMSCs. After MMSCs were cultured under normoxic (21% O_2_), hypoxic (1% O_2_) conditions or treated with LiCl (20 mM) or BIO (5 nM) for 3 days, detection of epithelial cell-related cell surface markers (E-cadherin or CK-18) was performed via flow cytometry. (a)-(b) Results of flow cytometry show that the number of epithelial cells was increased in hypoxia-cultured cells compared with those in normoxic conditions. Activating the Wnt/*β*-catenin pathway with LiCl or BIO attenuated the facilitatory effect of hypoxia on differentiation, as evidenced by a decreased percentage of E-cadherin and CK18-positive cells among LiCl- or BIO-treated cells. (c)-(d) Group data from flow cytometry. The data are shown as the mean ± SD. ^*∗∗∗*^*p* < 0.001, *n* = 3. (e) Expression of E-cadherin was detected by western blotting. The results show that 1% O_2_ increased and treatment with LiCl and BIO decreased the protein level of E-cadherin; ^*∗∗*^*p* < 0.01; *n* = 6. (f)–(h) Expression of CDH6, Aqp1, and OPN was detected by qRT-PCR; the result was consistent with that of E-cadherin; ^*∗*^*p* < 0.05; ^*∗∗*^*p* < 0.01; *n* = 3. Data are represented as mean ± SD.

We performed flow cytometry of MMSCs under hypoxic conditions (1% O_2_) in comparison to conventional cell culture conditions (21% O_2_) for 3 consecutive days. Many more E-cadherin- or CK18-positive cells were observed among cells exposed to hypoxia, compared with those subjected to normoxic conditions (E-cadherin: 30.1 ± 0.56% versus 10.6 ± 4.3%; CK18: 30.6 ± 2.4% versus 1.7 ± 0.6%, both *n* = 3, *p* < 0.001) (Figures 5(a)–5(d)). Additionally, we performed western blotting to explore the expression of E-cadherin in protein extracted from hypoxic and normoxic treated MMSCs. The protein level of E-cadherin was increased by hypoxic culture for 3 days (increased by 46 ± 5%, *n* = 6, *p* < 0.01) (Figure 5(e)). The RNA levels of CDH6, Aqp1, and OPN were also increased by hypoxic culture (Figures 5(f)–5(h)).

#### 3.5.2. Activation of the Wnt/*β*-Catenin Pathway Attenuated the Effect of Hypoxia on Differentiation of MMSCs

Flow cytometry showed that the percentage of E-cadherin and CK18-positive cells was reduced by treatment of LiCl or BIO compared with those exposed only to hypoxia (LiCl or BIO + hypoxia versus hypoxia: E-cadherin, 18.9 ± 0.85% and 15.0 ± 0.14% versus 30.1 ± 0.56%; CK18, 12.7 ± 1.77% and 9.25 ± 1.01% versus 30.6 ± 2.36%; *n* = 3, all *p* < 0.001) (Figures 5(a)–5(d)). This result was confirmed by reduced expressions of E-cadherin, CDH6, Aqp1, and OPN detected by western blotting or PCR (Figures 5(e)–5(h)), which suggested that activation of the Wnt/*β*-catenin pathway abrogated the differentiation of MMSCs induced by hypoxia.

### 3.6. Hypoxia Reduced the Stemness Phenotype of MMSCs by Inhibiting the Wnt/*β*-Catenin Pathway

#### 3.6.1. Hypoxia Reduced the Stemness Phenotype of MMSCs

**Figure 6:**
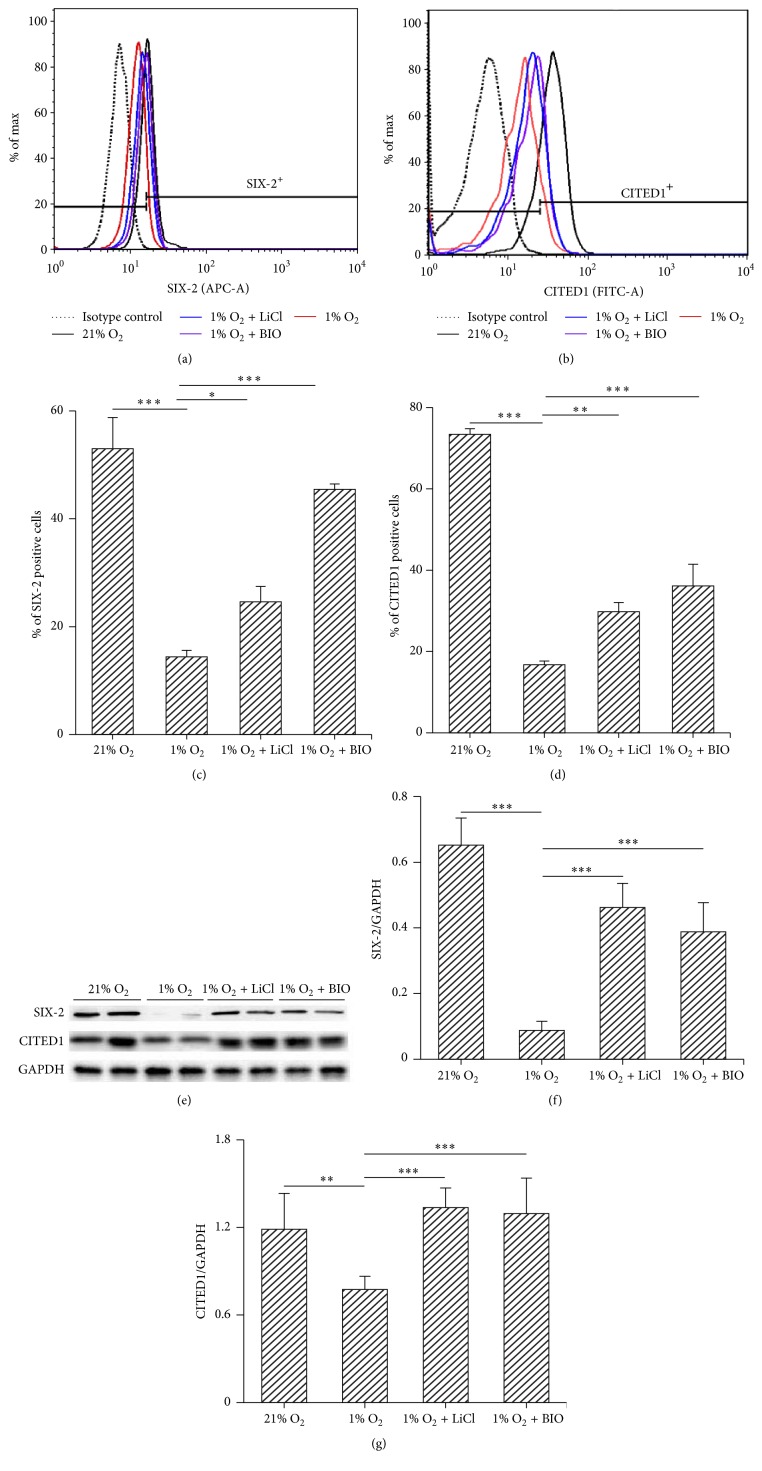
Stimulation of the Wnt/*β*-catenin pathway helps to maintain the stemness of MMSCs. MMSCs were treated as in Figure 4. Identification of stem cell-related cell surface markers (SIX-2 and CITED1) was performed via flow cytometry. (a)-(b) Results of flow cytometry show that the number of stem cells was decreased in hypoxia-cultured cells compared with those under normoxic conditions. Activating the Wnt/*β*-catenin pathway with LiCl or BIO attenuated the negative effect of hypoxia on stemness, as evidenced by an increased percentage of SIX-2 and CITED1-positive cells among LiCl- or BIO-treated cells. (c)-(d) Group data from flow cytometry. ^*∗*^*p* < 0.05; ^*∗∗*^*p* < 0.01; ^*∗∗∗*^*p* < 0.001; *n* = 3. Data are represented as mean ± SD. (e)–(g) Expressions of SIX-2 and CITED1 were detected by western blotting. The results show that treatment with LiCl and BIO increased the protein level of SIX-2 and CITED1; ^*∗∗*^*p* < 0.01; ^*∗∗∗*^*p* < 0.001; *n* = 6. Data are represented as mean ± SD.

To investigate the effect of hypoxia on stemness of MMSCs, flow cytometry was applied to count SIX-2- and CITED1-positive cells among isolated MMSCs that were subjected to hypoxic or normoxic conditions for 3 days. The result revealed that the percentage of SIX-2 and CITED1-positive cells was significantly decreased by hypoxic culture (hypoxia versus normoxia: 14.4 ± 1.2% versus 53 ± 5.8% and 16.8 ± 0.9% versus 73.4 ± 1.4%, *n* = 3, both *p* < 0.001) (Figures 6(a)–6(d)). To confirm the negative effect of hypoxia on stemness, western blotting was performed to detect the expression of CITED1 and SIX-2 in hypoxia-cultured cells. The expressions of CITED1 and SIX-2 were decreased by hypoxic culture for 3 days (Figures 6(e)–6(g)).

#### 3.6.2. Activation of the Wnt/*β*-Catenin Pathway Attenuated the Effect of Hypoxia on Stemness of MMSCs

Flow cytometry showed that the percentage of SIX-2 and CITED1-positive cells was increased by treatment of LiCl or BIO (LiCl or BIO + hypoxia versus hypoxia: SIX-2, 24.6 ± 2.85% and 45.47 ± 0.99% versus 14.4 ± 1.21%, *n* = 3, *p* < 0.05, *p* < 0.001; CITED1, 29.8 ± 2.25% and 36.1 ± 5.34% versus 16.77 ± 0.91%, *n* = 3, *p* < 0.01, *p* < 0.001, resp.) (Figures 6(a)–6(d)). This result was confirmed by elevated protein levels of SIX-2 and CITED1 detected by western blotting (Figures 6(e)–6(g)), which suggested that activation of the Wnt/*β*-catenin pathway mediated depressant effect of hypoxia on the stemness of MMSCs.

### 3.7. Hypoxia Inhibited Proliferation and Induced Apoptosis of MMSCs by Inhibiting the Wnt/*β*-Catenin Pathway

#### 3.7.1. Hypoxia Inhibited Proliferation and Induced Apoptosis of MMSCs

**Figure 7:**
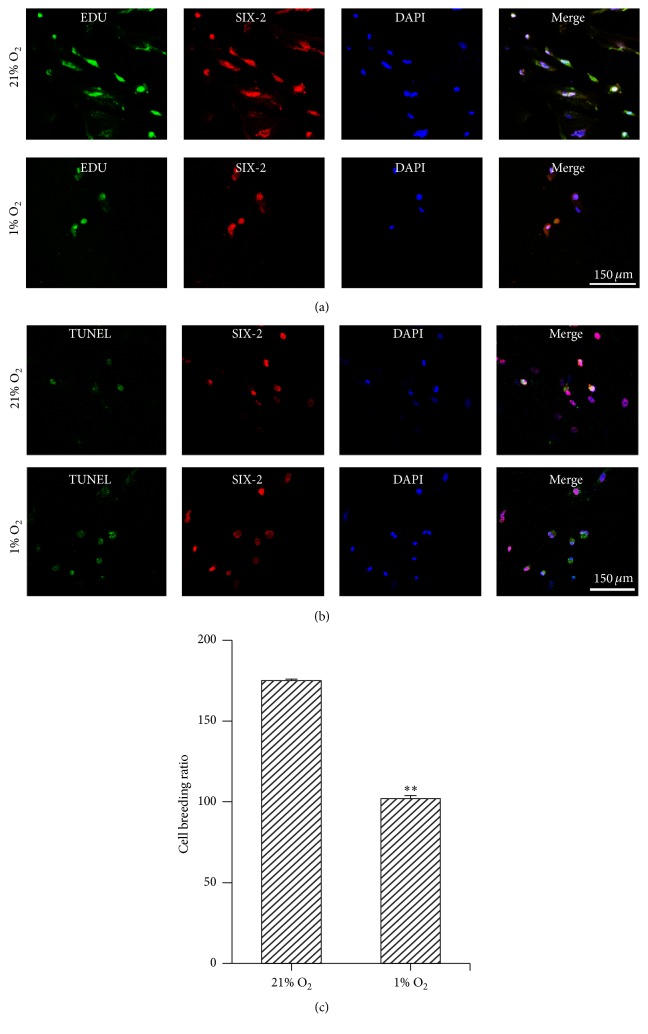
Hypoxia inhibited proliferation and elevated apoptosis of MMSCs. The MMSCs seeded in chamber slide system were incubated under hypoxic or normoxic condition for 3 days. (a) Proliferation of MMSCs was tested for by EdU staining. The number of EdU-positive cells was decreased under hypoxic conditions. (b) TUNEL staining showed that TUNEL-labelled SIX-2-positive cells were increased under hypoxic conditions. This also indicates that apoptosis mainly occurred in MMSCs. Bar is equal to 150 *μ*m. (c) The MMSCs seeded in 96-well plate were exposed to hypoxic or normoxic condition for 3 days. Breeding ratio of cells was measured by Alamar Blue staining; the data show that hypoxia depressed breeding of MMSCs. ^*∗∗*^*p* < 0.01; *n* = 6. Data are represented as mean ± SD.

**Figure 8:**
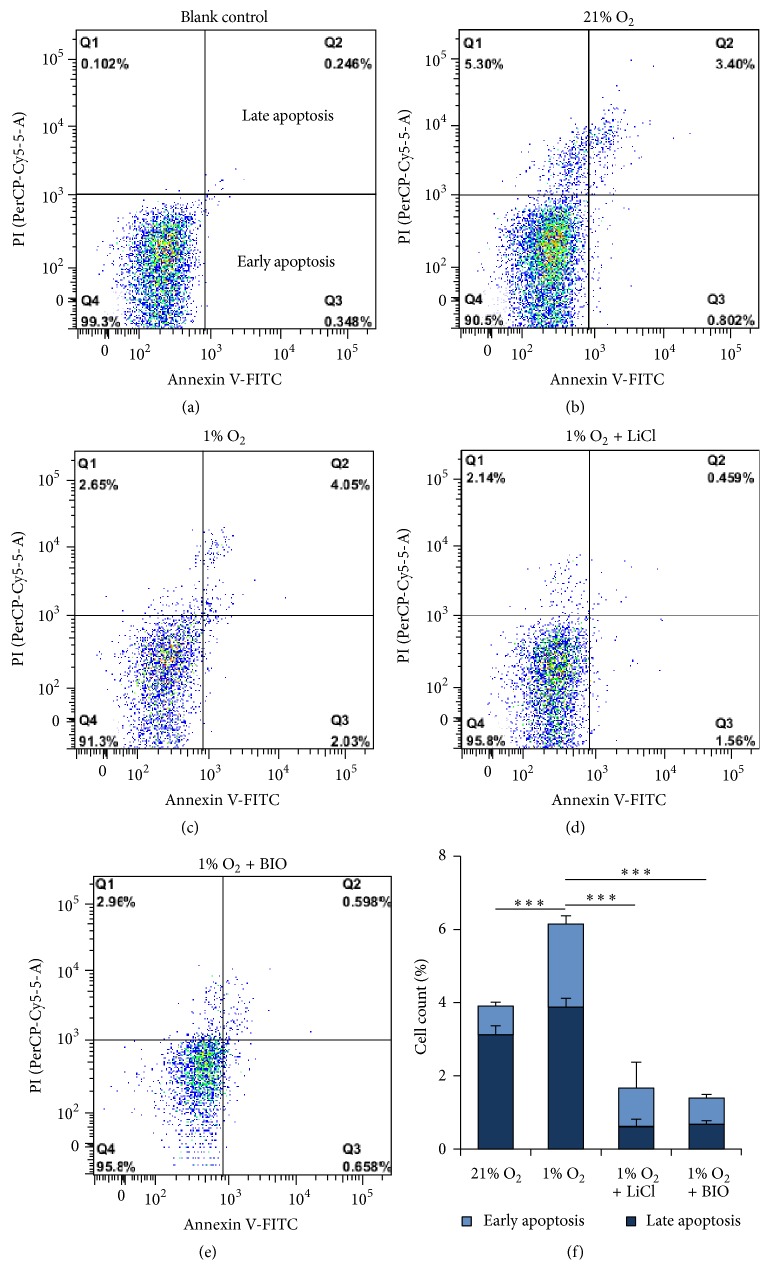
Stimulation of the Wnt/*β*-catenin pathway inhibited the apoptosis of MMSCs. Cells were treated as in Figure 4. The number of apoptotic MMSCs was detected by Annexin V-FITC/PI staining via flow cytometry. (a) Blank control. (b)–(e) Results of flow cytometry show that the percentage of cells undergoing early and late apoptosis (quadrants Q3 and Q2) among hypoxia-conditioned cells was larger than that in the control condition. Activating the Wnt/*β*-catenin pathway with LiCl or BIO decreased the percentage of apoptotic cells. (f) Group data from flow cytometry; ^*∗∗∗*^*p* < 0.001; *n* = 3. Data are represented as mean ± SD.

Proliferation of MMSCs was determined by Edu and Alamar Blue staining. MMSCs were suspended in serum-free media and plated onto chamber slides and 96-well plates. After hypoxic incubation for 3 days, the number of EdU-positive cells growing on the slide was counted, and the absorbance at the corresponding wavelength was measured for Alamar Blue staining. Our results showed that the number of EdU-positive cells and the breeding ratio were decreased in hypoxia-cultured MMSCs (breeding ratio under hypoxic versus normoxic conditions: 1.02 ± 1.92 versus 1.75 ± 0.82, *n* = 6, *p* < 0.01) (Figures 7(a) and 7(c)). Apoptosis of MMSCs was measured by TUNEL staining and flow cytometry. The results showed that the number of TUNEL-positive cells increased among hypoxia-cultured MMSCs (Figure 7(b)). As the flow cytometry showed, cells undergoing early and late apoptosis were both increased in hypoxia-cultured MMSCs (the early plus late apoptotic rate under hypoxic versus normoxic conditions: 6.14 ± 0.32% versus 3.91 ± 0.29%, *n* = 3, *p* < 0.01) (Figure 8).

#### 3.7.2. Activation of the Wnt/*β*-Catenin Pathway Attenuated the Effect of Hypoxia on Apoptosis of MMSCs

Flow cytometry showed that hypoxia-induced apoptosis of MMSCs was also inhibited by LiCl or BIO (LiCl or BIO + hypoxia versus hypoxia: 1.40 ± 0.20% and 1.67 ± 0.5% versus 6.14 ± 0.32%, *n* = 3, both *p* < 0.01) (Figure 8). These results indicated that stimulation of the Wnt/*β*-catenin pathway promoted the renewal of MMSCs.

Collectively, these findings support our hypothesis that hypoxia facilitates the differentiation and suppresses the renewal of MMSCs by inhibiting the Wnt/*β*-catenin signalling pathway.

## 4. Discussion

Hypoxia is a critical microenvironmental factor for normal embryonic development. Mammalian embryonic development occurs at low intrauterine O_2_ levels ranging from 2% to 9% [5]. Particularly in the embryonic kidney, oxygen tension can be as low as 1% [12]. Even in adult kidneys, which exhibit unique vascular supply, the kidney medulla and papilla experience oxygen tensions as low as 1%, a relatively hypoxic environment when compared to other tissues [30]. However, there is little evidence regarding the role of hypoxia in embryonic kidney development. To explore a more “physiological hypoxia” for the MMSCs, we compared the effects of different O_2_ tension (1%, 5%, and 7%) on differentiation and self-renewal of the MMSCs. We found that unlike 1% O_2_, 5% and 7% O_2_ had no effects on either differentiation or self-renewal of the MMSCs. Additionally, the metabolic/molecular biology studies performed on cell lines show that mitochondrial respiration can provide sufficient amount of energy under 1% O_2_ to permit cell proliferation [31]. Thus, we applied 1% O_2_ to research the effect of hypoxic condition on the MMSCs. We demonstrated that hypoxia promotes the differentiation and attenuates the stemness of MMSCs through blocking of the Wnt/*β*-catenin pathway.

The effect of hypoxia on the differentiation of stem cells varies among individual tissues. Hypoxia is known to promote cell differentiation and development in many tissues. It has been reported that hypoxia increases formation of terminal branches during tracheal development [32] and enhances the differentiation of mouse embryonic stem cells into distal lung cells [6]. Low oxygen increases the differentiation of neural progenitor/stem cells into mature neurons [7, 33, 34]. Hypoxic conditions increase the yield of cardiomyocytes from mouse embryonic stem cells [8]. In contrast, low O_2_ tension has been shown to inhibit the differentiation of human embryonic stem cells [35]. In the kidney, hypoxic conditions (5% O_2_) induce ureteric bud branching during kidney development in vitro organ culture [3, 5]. However, the effect of hypoxia on the differentiation of MMSCs was previously unclear. Our results revealed that hypoxia (1% O_2_) induced the differentiation of mesenchymal cell progenitors into epithelial cells, as evidenced by increased expression of the epithelial cell marker E-cadherin and an increased number of E-cadherin- and CK18-positive cells. It seems that hypoxia may induce ureteric bud branching through stimulating the differentiation of MMSCs. However, we found that hypoxic conditions (1% O_2_) inhibited ureteric bud branching in vitro organ culture [36]. The reason for this contradiction may be attributed to 1% O_2_ that we applied exceeding the tolerance of organ to hypoxia. Thus, further investigation will be needed to explore the effect of hypoxia on development of kidney.

In addition to inducing differentiation, maintaining stemness and proliferation of stem cells to preserve an available pool of undifferentiated progenitors is also important for development. Some reports have demonstrated that hypoxic culture (1–4% O_2_) maintains a higher level of undifferentiated human embryonic stem cells [4, 37]. A reduced-O_2_ environment has also been demonstrated to have positive effects on the maintenance of stemness of murine stem cells [38]. Moreover, a study has reported that hypoxia is not beneficial for maintaining human embryonic stem cells in the undifferentiated state [39]. Another study has demonstrated that mouse embryonic stem cells lose self-renewal activity under in vitro hypoxia [40]. Our present results showed that hypoxia exposure reduced the expression of SIX-2 and CITED1 and the number of SIX-2-positive cells. Additionally, proliferation was depressed and apoptosis was increased by hypoxia. Interestingly, apoptosis mainly occurred in SIX-2-positive cells but not in transformed epithelial cells. Because there are more MMSCs differentiated into epithelium and fewer regenerative cells to compensate in the stem cell pool, it is not surprising that the percentage of MMSCs was decreased after hypoxic culture. The results suggested that the significance of hypoxia for development lies in its potential to stimulate differentiation. There are probably other elements that help in promoting renewal of MMSCs in vivo, which will require further efforts to explore.

The development of the kidney is a developmental process in which mesenchymal to epithelial transition (MET) occurs. A mesenchymal stem cell pool responds to paracrine and autocrine factors that regulate whether cells remain within the niche or epithelialize. Among these factors, Wnt4 is considered to be a critical regulator in this process. Our investigation found that hypoxic culture inhibited Wnt/*β*-catenin, as evidenced by the attenuated level of Wnt4 and its downstream molecules *β*-catenin, LEF1, and Axin2. Previous investigations have revealed that stimulation of the Wnt/*β*-catenin signalling pathway in stem cells represses differentiation and maintains a state of self-renewal [41, 42]. To test whether hypoxia promoted the differentiation and inhibited the self-renewal of MMSCs by blocking the Wnt pathway, LiCl or BIO-conditioned media were used to stimulate Wnt signalling simultaneously with hypoxic exposure. Our results suggested that activation of the Wnt pathway neutralized the effect of hypoxia on MMSCs. In contrast, many studies have revealed that stabilization of *β*-catenin induces nephron differentiation [43]. However, a previous study has reported that LiCl elicits the early stages of epithelial differentiation, but the process fails to progress into the later stages of nephrogenesis [44]. Further, another study has observed that Wnt signalling acts transiently in inducing MET and that downregulation of *β*-catenin activity is essential for the fully epithelialized state of the renal vesicle [20]. In this context, the effects of Wnt/*β*-catenin on the differentiation of MMSCs may be distinct at different stages of development. Our data were collected from MMSCs harvested from embryonic kidney at embryonic days 18-19, which is near birth. A simple explanation is that hypoxia inhibited the Wnt/*β*-catenin pathway, whose activation blocks the differentiation of MMSCs at the late embryo stage in rats. Further studies may be required to explore the effects of hypoxia on the differentiation of MMSCs as well as on the Wnt pathway at early embryo stages.

Differentiation is a major hurdle for the successful translation of stem cell research to clinical applications. The ability of hypoxic preconditioning to induce the differentiation of stem cells toward mature cell lineages provides support for the application of hypoxic pretreatment before transplantation of stem cells. Indeed, accumulating evidence is suggesting that transplantation of hypoxia-preconditioned stem cells is more effective in tissue repair. It has been shown that the transplantation of hypoxia-preconditioned mesenchymal stem cells (MSCs) enhances vessel formation and skeletal muscle fibre regeneration after hindlimb ischemia and stimulates angiogenesis and neurogenesis after cerebral ischemia [16, 45]. It has also been demonstrated that intrastriatal transplantation of hypoxia-induced human MSCs (hMSCs) can more efficaciously ameliorate behavioural deficits of parkinsonian rats than normoxia-induced hMSCs [46]. Additionally, therapeutic applications of MSCs to treat heart failure, limb ischemia, and so forth are currently in Phase I (safety studies) or Phase II (proof of concept for efficacy in human patients) clinical trials [47, 48]. A Phase I clinical trial using MSCs for treating acute kidney injury (AKI) is ongoing [49]. Our previous experiments have revealed that hypoxic preconditioning enhances the therapeutic effects of bone marrow mesenchymal stem cells for treatment of AKI [50]. However, limitations still exist in the understanding of how hypoxic-preconditioned MSCs exert their renoprotective effects. Our current results indicate that the elevated therapeutic activity of MSCs may be attributed to the ability of hypoxia to promote differentiation. Although hypoxia increased apoptosis, the overall percentage of cells undergoing apoptosis is not high, being lower than 7% even under hypoxia. Thus, we concluded that the therapeutic potential of MMSCs could be enhanced by hypoxic precondition.

In summary, the observations herein further our understanding of the effects of hypoxia on kidney development by demonstrating that hypoxic conditions stimulate the differentiation of MMSCs by inhibiting canonical Wnt signalling. Additionally, this study aids in understanding the therapeutic potential and possible clinical impact of hypoxia-preconditioned MMSCs.

## Figures and Tables

**Table 1:** PCR primer sequence and size.

Gene name	Forward (5′-3′)	Reverse (5′-3′)
Wnt4	CTGGAGAAGTGTGGCTGTGA	AAAGGACTGTGAGAAGGCTACG
*β*-Catenin	CTTACGGCAATCAGGAAAGC	GACAGACAGCACCTTCAGCA
LEF-1	GCATCCCTCATCCAGCAA	GGCTCCTGTTCCTTTCTCTGT
Axin2	ACGGAATACGAAAGGCACAG	ACGCTCACTCTCCAACATCC
SIX-2	GCCAAGGAAAGGGAGAAC	CTGTGTAGGGAAGGCAACC
PKG1	AAGACGGCAAGCATGAAGCT	CCCTTCTGTCCCTGTAAAGGTTT
Glut1	GCTTCCTGCTCATCAATCGT	CTGCCGACCCTCTTCTTTC
CDH6	ATGACAATCCTCCTCGCTTC	TTTCTCCCACATCAGCATCA
Aqp1	CCGCAACTTCTCAAACCACT	CATCCAGGTCATACTCCTCCA
OPN	AAGCGTGGAAACACACAGC	TTTGGAACTCGCCTGACTG
GAPDH	GGGTGTGAACCACGAGAAAT	ACTGTGGTCATGAGCCCTTC
